# Phase Transitions and Electrochemical Properties of Ionic Liquids and Ionic Liquid—Solvent Mixtures

**DOI:** 10.3390/molecules26123668

**Published:** 2021-06-16

**Authors:** Carolina Cruz, Alina Ciach

**Affiliations:** Institute of Physical Chemistry, Polish Academy of Sciences, 44/52, 01-224 Warsaw, Poland; ccruz@ichf.edu.pl

**Keywords:** ionic liquids, ionic liquid-solvent mixtures, demixing phase transition, effects of confinement in ionic systems, differential capacitance, electrical double layer, supercapacitors

## Abstract

Recent advances in studies of ionic liquids (IL) and ionic liquid–solvent mixtures are reviewed. Selected experimental, simulation, and theoretical results for electrochemical, thermodynamical, and structural properties of IL and IL-solvent mixtures are described. Special attention is paid to phenomena that are not predicted by the classical theories of the electrical double layer or disagree strongly with these theories. We focus on structural properties, especially on distribution of ions near electrodes, on electrical double layer capacitance, on effects of confinement, including decay length of a dissjoining pressure between confinig plates, and on demixing phase transition. In particular, effects of the demixing phase transition on electrochemical properties of ionic liquid–solvent mixtures for different degrees of confinement are presented.

## 1. Introduction

Ionic liquids (ILs) and IL–solvent mixtures (ILS) have turn into the research focus in electrochemistry due to their intriguing properties, such as exceptional electrochemical and thermal stability and low vapor pressure that make them attractive materials for several applications [1,2]. Namely, ILs are used in electrochemical reactions, as lubricants for micro and nanodevices [3], as extraction liquids for metals purification, colloids, and biomass, etc. [2]. One of the key applications is in the field of energy generation and storage. Deep knowledge of ILs and IL-solvent mixtures is crucial for developing devices such as supercapacitors, batteries, solar cells, and fuel cells.

For various industrial applications of fluids and fluid mixtures, confinement effects play a very important role [4]. ILs under confinement exhibit remarkable properties in energy storage applications [5,6,7,8], capacitive deionization [9,10,11], and heat-to-energy conversion [12,13,14]. For instance, pores in the subnanometer scale filled with an electrolyte provide the highest feasible capacitance [15,16,17] and stored energy [18] but with slow dynamics [19,20,21,22]. Using neat ILs in micro and mesopores improves the stored electrical energy [23]. Even so, neat ILs show slow dynamics, and to improve their conductivity, ILs are mixed with solvents such as water or acetonitrile [24,25,26,27], allowing to speed up the charging kinetics [28].

Most of the studies of IL-solvent mixtures confined by micro and mesopores have been focused on thermodynamic states far from phase transitions. Nevertheless, fluids under confinement show an interesting physics close to state transformations. Typical examples are the wetting transition and the capillary condensation [29,30,31] which have several practical applications, notably in the determination of the pore-size distribution of micro and mesoporous materials [32,33,34].

IL-solvent mixtures can undergo a demixing phase transition, in analogy to charge-neutral liquids, and in recent experiments, phase diagrams for various ILs and various solvents have been determined. In particular, organic IL with a polar solvent like water can phase-separate at room temperature, and the critical point of this phase separation is of the order of 400 K. The studies of the phase separation in IL-solvent mixtures concerned mainly the bulk systems. The investigation of the effect of proximity to the demixing phase transition on electrochemical properties of confined IL-solvent mixtures has started only very recently [35,36,37].

In this review, we describe the classical theories of electrical double layers briefly and define the fundamental quantities in Section 2. In Section 3, the IL and IL-solvent mixtures are characterized, with the focus on their structure (Section 3.2) capacitance (Section 3.3) and effects of confinement (Section 3.4). In Section 4, the demixing phase transition is discussed, and the experimental results for the phase diagrams in IL-solvent mixtures are presented. Bulk properties are summarized in Section 4.1, and in Section 4.2, confinement-induced phase transitions are described. We pay special attention to the effects of proximity to the demixing phase transition on electrochemical properties of IL-solvent mixture (Section 5). Results for the IL-solvent mixtures in contact with a single electrode are presented in Section 5.1, and the case of a slit-shaped pore is discussed in Section 5.2.

## 2. Classical Theories of Electrical Double Layers

Electrical double layer (EDL) results from the formation of a ‘cloud’ rich in ions with a charge of a sign opposite to the surface charge (known as counterions) and poor in ions of the same sign (known as coions). The EDL width is determined by the interplay between the ions’ thermal motion, which tends to homogenize their distribution, and the Coulombic attraction of the counterions to the surface and repulsion of the coions from it [38,39].

Helmholtz proposed the first model for describing EDLs in 1853. In his model, the surface charge was neutralized by counterions which were assumed to be adsorbed at the electrode surface [40]. This model can be understood as a single layer of ions driven to the surface by its electric field, which is completely screened by the ionic layer.

In the early 20th century, the achievements of Gouy, Chapman, Debye, Hückel, and Langmuir developed the classical theory of electrolytes and the so-called Poisson-Boltzmann (PB) model gathered such achievements [41,42]. In the PB model, it is assumed that ions can be modeled as mobile point-like charges immersed in a dielectric continuum characterized by a dielectric constant [38,40,41,43]. In the Gouy–Chapman theory, it is considered that ions have no physical limitations to approach the surface. However, Stern modified this model proposing that ions have a finite size and they are not precisely at the surface but at some distance away from it [2]. Thus, there is a compact layer of counterions known as the Stern layer and a diffuse layer. In the Gouy–Chapman–Stern theory (GCS), the potential drops down linearly in the Stern layer and then exponentially through the diffuse layer [2]. Within the linearized PB (Debye–Hückel approximation), the decay length of the potential is equal to the Debye screening length

(1) $$\lambda_{D}={\left(4\pi \rho_{b}\lambda_{B}\right)}^{-1/2},$$

where $\rho_{b}$ is the ion density, and $\lambda_{B}$ is the Bjerrum length given by

(2) $$\lambda_{B}=e^{2}/\left(k_{B}T\epsilon \right),$$

where ϵ is the dielectric constant, $k_{B}$ the Boltzmann constant, and *T* temperature. The Debye screening length characterizes the thickness of the EDL, while the Bjerrum length is the distance between the ions at which the Coulomb potential equals the thermal energy $k_{B}T$.

One significant effect which is not considered in the GCS theory is the excluded volume interactions that take into account the physical size of ions. The excluded volume interactions restrain the counterions adsorption and influence the charge density in the EDL [44]. Although ion’s charge correlations and fluctuations are neglected, the application of the PB model to EDLs still forms the basis of the understanding of electrolyte solutions. For instance, it predicts the well-studied U-shaped EDL differential capacitance C(U) as a function on the applied potential *U* [41], where

(3) $$C\left(U\right)=\frac{dQ}{dU}.$$

In the case of monovalent ions, the charge accumulated in the EDL is

(4) $$Q=\int_{0}^{\infty }ec\left(z\right)dz$$

with

(5) $$c\left(z\right)=\rho_{+}\left(z\right)-\rho_{-}\left(z\right),$$

$\rho_{+},\rho_{-}$ denoting the densities of cations and anions, and *e* denoting the elementary charge. The energy stored in the EDL is given by the formula

(6) $$E\left(U\right)=\int_{0}^{U}C\left(u\right)udu.$$

The polarizability of solvent and ions may have a significant influence on the EDLs’ structure and properties. It has been reported that ϵ depends on temperature, especially for polar solvents [45,46,47]. In addition, the change of ϵ close to the surface affects the results quantitatively [48,49,50,51]. To capture generic effects, ϵ is often assumed to be temperature-, density- and position independent [50,51,52,53,54,55,56,57,58,59]. Theories with the assumption of constant ϵ cannot be accurate on the quantitative level but capture generic effects and qualitative behavior.

## 3. Ionic Liquids

### 3.1. General Properties

Room-temperature ionic liquids (RTILs), or simply ionic liquids (ILs), are commonly defined as materials composed of an organic or inorganic anion and an organic cation that melt below 100 °C 150 °C. In conventional electrolytes, ions are described as small and almost round with a uniform charge density, immersed in a solvent and interacting with each other by Coulomb forces [2]. However, in ILs, ions are not round, and their molecular charge densities are non-uniform. Additionally, there is no solvent unless it is added or absorbed from the environment. Due to these complex features, ILs are liquids at room temperature, and the effect of short-range interaction between ions, size and shape of the ions, and charge distribution are crucial factors for describing their properties in bulk and at interfaces.

**Figure 1:**
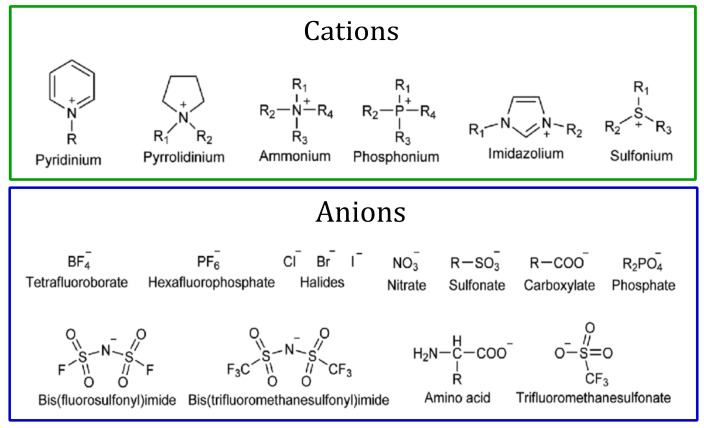
Typical cations and anions in ionic liquids. Typical basis of the cations are ammonium, imidazolium, sulfonium, piperidinium, and pyridinium ions. Halides, tetrafluoroborate, nitrate, sulfonate, carboxylate, phosphate, amino acid, among others, serve as a base of the anions for the preparation of ILs.

The first IL (ethylammonium nitrate) was reported in 1914 by Paul Walden, and this work determined the starting point of the first generation of ILs. The first generation of ILs is characterized by cations of large volumes, such as 1,3-dialkyl-imidazolium or 1-alkylpyridinium, and anions are based mostly on halogen aluminate (Al^+3^). This generation, however, was unstable with respect to air and water. For the second generation of ILs, the aim was to obtain air- and water-stable ILs based on the 1-ethyl-3-methylimidazolium cation and [CH3CO2]^−^, [NO3]^−^ and [BF4]^−^ as alternative anions. The resulting ILs were easy to manipulate and awaken scientific attention due to their particular properties. The third generation of ILs exhibits tunable chemical and physical properties depending upon the desired applications [2,60,61,62]. Typically, ILs structure combines organic cations with either organic or inorganic anions. Figure 1 shows some common cations and anions to obtain ILs [2,60,63].

According to their synthesis route, ILs are usually classified as aprotic ionic liquids (APILs) and protic ionic liquids (PILs) [60]. PILs are obtained via proton transfer from a Brønsted acid to a Brønsted base. PILs usually exhibit higher conductivity and melting points compared to APILs [64].

Regarding their cation segment, ILs are commonly classified into four types: (1) alkylammonium-, (2) dialkylimidazolium-, (3) phosphonium-, and (4) *N*-alkylpyridinium-based ILs. Ammonium-based ILs exhibit electrochemical cathodic stabilities, low viscosities, and low melting points; thus, they have been used as electrolytes in electrochemical devices. Imidazolium-based ILs have been widely studied. They are easily synthesized and have remarkable stability under oxidative/reductive conditions. Therefore, imidazolium-based ILs are used as a catalyst to improve the reaction yield and the chemoselectivity of several organic reactions. However, it is important to note that the selection of this type of IL as a cosolvent for a reaction under basic conditions should be carefully considered to avoid undesired side reactions. For instance, in [65] the authors found that in a base-catalyzed Baylis–Hillman reaction in the presence of imidazolium-based ILs, the catalyst was deactivated due to a side reaction involving the imidazolium-based IL [62]. Pyridinium-based ILs are highly stable, and the catalytic role of this type of ILs is outstanding in the synthesis of some pharmaceutical agents [62]. Phosphonium-based ILs are the most thermally stable compared to the imidazolium- and pyridinium-based ILs. Thus, phosphonium-based ILs are suitable for reactions at higher temperatures (more than 100 °C). In [66], phosphonium-based ILs have been used for CO2 capture.

ILs are versatile and have a unique combination of properties, meaning that, by mixing them and choosing the appropriate cation-anion combinations, it is possible to tune specific properties. The selection of the cation defines the stability of the IL, whereas its functionality is usually controlled by choosing the anion. In a general view, most ILs remain in the liquid state up to temperatures in the range 200 °C 300 °C (at atmospheric pressure), in contrast to water or common organic solvents that evaporate at lower temperatures [67]. Additionally, ILs have low volatility as a result of the strong ion–ion interactions [68]. Although ILs are stable at high-operation temperatures in energy storage devices [69,70], in some cases, undesired electrochemical reactions involving ILs could be activated [71]. Furthermore, it is important to note that, for temperatures higher than 200 °C 300 °C, ILs undergo thermal decomposition that may generate flammable and toxic gases [72,73].

Most of the ILs are electrochemically stable at charged interfaces. In applications such as batteries, supercapacitors, electrocatalysis, and electrodeposition, the ILs must remain as stable as possible in the potential range. The electrochemical window (EW), defined as the voltage range in which a substance is neither oxidized nor reduced, is a measure of such stability and is one of the most important features to be identified for solvents and electrolytes. The large EW of some ILs allows achieving high electrode charge densities that are inaccessible for conventional electrolytes [74,75,76].

As mentioned in Section 1, an important limitation of ILs as supercapacitor electrolytes is their high viscosity since it affects ionic mass transport. At low temperatures, the commercially available ILs do not possess high conductivity. Thus, various strategies have been implemented to decrease the relatively high viscosity of ILs and enhance their conductivity [77,78,79,80,81]. For instance, McEwen et al. [82] reported a conductivity enhancement of ethylmethylimidazolium hexafluorophosphate (EMImPF6) when mixed with some organic solvents such as propylene carbonate (PC), dimethyl carbonate (DMC), diethyl carbonate (DEC), ethylene carbonate (EC), and ethyl methyl carbonate (EMC). In a succeeding work, McEwen et al. [83] investigated a series of IL-solvent mixtures. The authors found that the mixture of acetone and EMImPF6 mixture exhibits the highest conductivity. Although such enhanced conductivity is similar to the case of acetonitrile electrolytes, there is a safety matter since acetonitrile electrolytes are toxic at higher temperatures [83,84]. Likewise, Jarosik et al. [85] have investigated ILs-solvent mixtures such as 1-ethyl-3-methyl imidazolium trifluoromethane sulfonate triflate (EMImTf) mixed with 1-ethyl-3-methylimidazolium bis(trifluoromethanesulfonylimide) (EMImNTf2). The authors found that those mixtures exhibit higher conductivity with respect to the conductivity of their respective neat ILs.

### 3.2. Structural Properties

Due to the wide variety of cation/anion pairs available to obtain ILs and the substantial differences in ion dimensions, characterization of the physicochemical properties of ILs still seems to be challenging. Nevertheless, although it is difficult to accurately predict IL properties from the molecular structure, some insight into IL properties has been gained [86]. For instance, Chiappe and Pieraccini [87] discussed the physicochemical properties of the most used ILs in terms of the structure of the respective anion and cation forming the ILs. To mention some examples, the melting-point of a series of imidazolium-based ILs decreases when the size and asymmetry of the cation increase. However, it is more complex to analyze the anion effect. For imidazolium-based ILs composed of structurally similar anions, bis(trifyl)imide ([Tf2N]^−^) exhibits lower melting-point compared to triflate ([TfO]^−^), and such a difference has been attributed to the electron delocalization and the hydrogen bonding absence. In the case of thermal stability, Ngo et al. [88] have proposed that, regarding the anion, the thermal stability is [PF6]^−^ > [Tf2N]^−^ ∼ [BF4]^−^ > halides. Concerning the cation size, there is no significant effect when increasing the size from 1-butyl to 1-octyl ([BMIM]^+^ to [OMIM]^+^). Regarding the viscosity, Xu et al. [89] studied some orthoborates-based ILs and found that in the case of ILs having the [BMIM]^+^ cation, the viscosity decreases with increasing anion volume. The authors attributed such behavior to the fact that the counterparts, positive and negative, become more even in size.

In addition to variety of sizes and shapes of the ions, ILs have revealed an exciting interplay among various interactions, from weak and nonspecific forces, such as van der Waals and dispersion forces, to strong and specific interactions (Coulombic forces, hydrogen bonds, halogen bonds, dipole-dipole interactions, etc.). Such a diversity of inter and intramolecular forces favors the formation of heterogeneous microstructures and liquid morphologies in bulk and near interfaces [90,91]. Furthermore, a significant number of ILs have polar and apolar components, and thus, they can be treated as segregated liquids with polar and apolar domains [91]. For this reason, ILs exhibit dynamic heterogeneity, meaning that there is a distribution of relaxation rates for molecules in different local environments [92]. Sha et al. [93] performed molecular dynamics simulations to study the heterogeneous dynamics in local IL heterogeneous structures and found that such heterogeneous dynamics come from the strong association between cations and anions, resulting in a slow dynamic behavior in polar regions. In contrast, the accelerated dynamic behavior in apolar regions is due to the weaker vdW interactions between alkyl chains.

ILs with and without dilution may exhibit self-assembly and non-monotonic variation of the EDL with respect to the concentration [43,94]. One intriguing behavior is a strong layering effect at the interface observed for the first time by atomic force microscopy (AFM) [95,96,97], and then confirmed by high-energy X-ray reflectivity [98,99]. Although layering is present for all the ILs, Perkin et al. [100] have reported considerable differences in the distance between the successive layers of adsorbed ions. The length characterizing patterns such as alternating cation-anion monolayers or tail-to-tail cation bilayers is determined by the sizes and shapes of the ions; therefore, it takes significantly different values in different ILs.

Atomistic simulations of IL-alcohol mixtures show exponentially damped charge oscillation in the direction perpendicular to the electrode that follows very well the formula

(7) $$c\left(z\right)=A_{c}\exp\left(-z/\lambda_{s}\right)\sin\left(k_{0}z+\theta \right)$$

already for $z>2\pi /k_{0}$ [101]. The simulations were performed for pure 1-butyl-3-methylimi-dazolium tetrafluoroborate ([BMIM][BF4]) as well as for [BMIM][BF4] with water or amphiphilic solvents such as methanol and ethanol. Exponentially damped charge oscillation was observed earlier in simulations of oppositely charged hard spheres with equal diameters [49], in molecular dynamics simulations of a coarse-grained model of ions and solvent [102], and in recent theory [103]. It seems that the charge ordering near an electrode can be described by the above formula, independently of the size and shape of the ions. In directions parallel to the electrode, the formation of hexagonal or striped arrangement of coions in a layer near the electrode was observed in atomistic simulations of the same IL-alcohol mixtures [104].

### 3.3. Electrical Double Layer Differential Capacitance

One of the most intriguing features of ILs is the complicated shape of the double layer capacitance as a function of the electrostatic potential. Most of the capacitance-voltage curves have one or two maxima depending on the type of IL, temperature, and the nature of the electrodes [105], rather than the U-shaped capacitance predicted by the classical theories. However, the classical models of EDLs are only valid on the basis of dilute-solution approximation (concentrations below 0.01 M) [43]. In the case of ILs and IL–solvent mixtures, the concentration of ions is high, and the classical models are no longer valid. In particular, the ion sizes become significant [106,107,108,109]. Indeed, EDLs theories have manifested that excluded volume interactions are essential to properly describe the structure of the EDL with ILs [44,52,53,54,55,56,58,110,111].

To get a deeper understanding of the differential capacitance shapes, Kornyshev and Fedorov et al. [2,50] proposed a mean-field theory that provides a classification in terms of packing (or volume fraction) of ions. When there are some voids or solvent molecules at the EDL, then once the voltage increases, the solvent molecules are expelled out, and the EDL will be filled with ions. As a consequence, the capacitance will increase, exhibiting the camel shape, i.e., a minimum at the potential of zero charge, denoted by PZC (a metallic electrode carries a charge density whose magnitude depends on its potential. The specific potential at which no charge is carried is called the potential of zero charge. This is a distinctive quantity for a given metal/solvent interface, and it is independent of the ions in the case in which there is no specific adsorption) and two symmetric maxima [35]. The camel-shaped capacitance is a signature of dilute electrolytes and has been extensively studied [49,52,53,54,55,59,112,113,114,115]. On the other hand, if there are almost no voids or few solvent molecules, the counterions will start to accumulate at the electrode, and the EDL will get thicker. Consequently, in the case of large ionic density, the capacitance will take the bell shape, with a single maximum at the PZC [35,49,52,53,54,55,59,111,112,113,114,115,116,117].

Temperature plays an essential role within the structure and capacitance of EDLs as well, but contradictory results have been found in the literature. An agreement on whether or not capacitance will increase or decrease with temperature needs to be reached. In accordance with the Guy–Chapman theory, the capacitance should decrease as the temperature increases. However, the experiments demonstrated the opposite trends [116,118,119,120] too. For example, Silva et al. [118] studied the ionic liquid 1-Butyl-3-methylimidazolium hexafluorophosphate ([BMIM][PF6] with 1-butyl-3-methylimidazolium, [BMIM]^+^, as cation) and three different electrodes. The main finding was that the differential capacitance increases along with the temperature for all the considered potentials. Lockett et al. [116] encountered the same trend for glassy carbon electrodes in contact with imidazolium-based ionic liquids. More meticulous theoretical studies indicated that both trends could occur [57,121,122], although the origin of this behavior lacks consensus. For example, Holovko et al. [121] suggested that the capacitance increases because of decreasing inter-ionic interactions and weaker ion associations. Chen et al. [57], on the other hand, proposed that the strength and extent of van der Waals interactions predominantly determine the EDL’s temperature dependence. Intriguingly, it was established that temperature variations could also induce the transition between the camel and bell-like capacitance [57,115], with the bell shape appearing at high temperatures due to ‘ion pairs’ breaking and therefore stronger screening [57].

### 3.4. Effects of Confinement

ILs have become promising candidates for many applications in energy storage devices such as dye-sensitized solar cells, supercapacitors, and fuel cells, mainly when confined to pores of nanoscopic or mesoscopic dimensions [115,123,124,125]. The understanding of the structure of the ions on electrode surfaces as well as the properties of the electrical double layers (EDLs) displayed by the interactions between ions and polarized electrodes are of paramount importance and have been the subject of many theoretical [101,103,104] and experimental works [126].

A deep understanding of the ILs behavior inside pores is still needed, and one of the features to analyze is whether the ions will locate preferentially at the surface or tend to keep a bulk-like structure [94]. Nuclear magnetic resonance (NMR) experiments performed on ILs confined in porous carbon electrodes revealed that the ILs spontaneously wet the carbon pores in the absence of any applied potential. However, when applying potential, charging occurs by adsorption of counterions and desorption of coions from the pores [127]. Similar results were reported from simulations of slit-shaped pores by performing molecular dynamics [128,129,130], or implementing classical density functional theory approaches [125,131].

Specifically, in the theoretical formulations, Pizio et al. [125] found that the differential capacitance exhibits an oscillatory behavior as a function of the pore width, and the magnitude of the oscillations decreases when the electrostatic potential increases. Furthermore, the capacitance reaches a minimum value for narrower pores. The authors suggested that the capacitance oscillations could be related to the interference of the EDLs formed at the pore walls. Similar behavior was also found by Jiang et al. [131]. In this work, a classical density functional to describe an IL electrolyte inside a nanopore was implemented. The results reveal that the capacitance oscillations decay when the pore size increases from one to many times the ion diameter. Moreover, a peak capacitance was found due to the constructive interference of the EDLs at each wall. These results could imply that the optimal capacitance can be reached by controlling the pore width.

An interesting difference between IL-solvent mixtures and dilute electrolytes is the decay of the disjoining pressure between plates confining the electrolyte. The disjoining pressure decays exponentially for the increasing distance between the plates, with the decay length $\lambda_{s}$ that is supposed to be equal to the bulk correlation length. In dilute electrolytes, $\lambda_{s}=\lambda_{D}$, in perfect agreement with theoretical predictions. As shown in recent experiments [132,133,134,135], however, in concentrated electrolytes and IL-solvent mixtures $\lambda_{s}∼l_{B}\rho_{b}$. The above and Equation (1) lead to the scaling relationship $\lambda_{s}/\lambda_{D}∼{\left(a/\lambda_{D}\right)}^{3}$, where *a* is the ion diameter [134]. The scaling behavior was observed for a number of simple salts (NaCl, LiCl, Kl, CsCl) in water, and for 1-butyl-1-methylpyrrolidinium bis[(trifluoromethyl)sulfonyl]imide ([C4C1Pyrr][NTf2]) in a number of solvents (propylene carbonate, dimethyl sulfoxide, acetonitrile, anhydrous benzonitrile), as well as for pure ILs [C2mim][NTf2] and [C3mim][NTf2] (in the latter case, the temperature dependence was verified). A scaling law for the screening length has been confirmed in theories based on different assumptions [136,137,138,139,140,141] and in all-atom molecular dynamics simulations [142,143]. The scaling exponents, however, appeared to be significantly lower than the experimentally measured one. The relation $\lambda_{s}∼l_{B}\rho_{b}$ was found for the decay length of the charge–charge correlation function in [141], where the significant role of the local variance of the charge density was demonstrated. The charge correlations exhibit an oscillatory decay, however, while the experiments show monotonic decay of the disjoining pressure at large separations. The puzzle of a large range of the disjoining pressure between plates confining concentrated IL-solvent mixtures remains unsolved.

## 4. Demixing Phase Transitions

### 4.1. Phase Transitions in the Bulk

At a first-order phase transition, an initially one-phase system separates into two different coexisting phases (e.g., vapor-liquid or liquid-liquid coexistence). Phase separation processes in a binary mixture are often studied under the assumption of fixed total number of particles, $N=N_{1}+N_{2}$. In this case, the appropriate thermodynamic potential is the the semi-grand thermodynamic potential given by $\Omega =U-TS-\mu N_{1}$, where U is the internal energy, *S* the entropy, and $\mu =\mu_{1}-\mu_{2}$ is the difference between the chemical potentials of the two components. In the particular case of ILS, $N_{1}=N_{IL}=N_{+}+N_{-}$ and $N_{IL}/V=\rho =\rho_{+}+\rho_{-}$ denotes the IL density.

**Figure 2:**
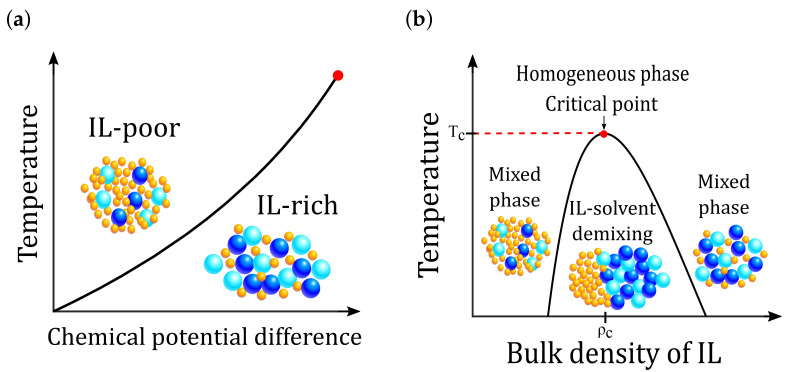
Schemes of an IL-solvent mixture phase diagrams. (**a**) Scheme of an IL-solvent mixture phase diagram in the temperature-chemical potential plane. Below $T_{c}$ (shown by the red circle), two phases coexist along a saturation chemical potential $\mu_{sat}$. One phase is rich in IL, and the other phase is rich in the solvent. Light blue spheres denote cations, while blue spheres denote anions. Solvent molecules are denoted by yellow spheres. (**b**) Scheme of an IL-solvent mixture phase diagram in the temperature-concentration plane. Above the critical temperature $T_{c}$, the system is homogeneous for any temperature and bulk composition of IL. Below $T_{c}$, the mixture phase separates into one phase rich in IL and the other phase one rich in the solvent. The critical composition of IL is denoted by $\rho_{c}$.

Figure 2a is a schematic representation of a phase diagram of IL-solvent mixtures in the chemical potential–temperature plane. The coexistence line ends at an upper critical point marked by the red circle. Figure 2b is a schematic representation of the phase diagram in the concentration-temperature plane. Above the critical temperature $T_{c}$, the system is homogeneous at any temperature and bulk composition of IL. However, below $T_{c}$, the homogeneous IL-solvent mixture becomes unstable for a certain range of concentration, and it will spontaneously phase-separate into two phases, one phase rich in IL and the other one rich in the solvent. These two phases coexist along a saturation composition curve, which terminates at a bulk critical point: $\left(\rho_{c},T_{c}\right)$, where $\rho_{c}$ is the critical composition of IL.

At the critical point, a phase transition occurs due to the competition between the internal energy U that favors order and the entropy *S* of the system, which benefits disorder. Depending upon the temperature value, one of the terms dominates [144]. For T→∞, the entropic contribution of the thermodynamic potential dominates, leading to the stability of the disordered phase. However, by decreasing temperature, the system starts ordering upon approaching an upper critical point, and droplets or correlated regions of the same type of molecules are formed. For $T\to T_{c}$, the size of the correlated regions, the so-called correlation length ξ, increases and diverges at the critical point ($T=T_{c}$).

The existence of the critical point associated with the separation into ion-poor and ion-rich phases was first predicted by Stell et al. [145] for purely Coulombic interactions between charged hard spheres of equal diameter (so-called restricted primitive model, RPM). In subsequent theoretical, simulation and experimental studies the existence of the phase separation in the RPM-like systems was confirmed [146,147,148,149,150,151,152,153,154]. The critical density of ions at the Coulombic critical point is low, however, and this phase transition is relevant for dilute electrolytes. When specific non-Coulombic interactions play a significant role, as is the case, for example for organic ions and polar solvents like water, then the phase separation can be induced by these specific interactions [148,155]. In this case, the critical density is larger, and this type of phase transition is relevant for IL-solvent mixtures.

The experimental phase diagrams for various IL-solvent mixtures are similar to the schematic diagram shown in Figure 2b [156,157]. It has been reported that various imidazolium-tetrafluoroborate ILs in arenes, water, and alcohols have critical temperatures within the range 300 K to 400 K, and critical mole fractions from 0.02 to 0.125 [157]. The intensively studied 1-hexyl-3-methylimidazolium tetrafluoroborate (C_6_mim-BF_4_) exhibits the critical temperature $T_{c}\approx 326$ K, and the critical mole fraction $x_{c}\approx 0.125$ in alcohol (C_6_OH), while in water $T_{c}\approx 331$ K and $x_{c}\approx 0.04$ [157]. Aqueous bistriflimide (TFSI)-based ILs show higher critical concentrations (between 0.2 and 0.3), with the critical temperature in the range from 400 K 420 K [156]. The small density of ions at the critical point in the above experimental systems signals that the phase separation is induced primarily by the Coulomb interactions, while the separation driven mainly by the specific, short-range interactions takes place when the critical mole fraction is $x_{c}>0.1$.

### 4.2. Confinement-Induced Phase Transitions

Fluids under confinement can undergo a dramatic change in their equilibrium and dynamical properties. The behavior of a confined fluid is directly related to the pore morphology, topology, and the magnitude of fluid–surface and fluid–fluid interactions. As a result, new phase transitions such as wetting, pre–wetting, filling transitions in wedges, and capillary condensation, may take place [158,159,160,161].

Furthermore, when the pore size is of an order of magnitude comparable to the range of the intermolecular forces, there will be a reduction of the number of nearest-neighbor molecules felt by the confined molecules. This effect leads to a shift in phase coexistence curves and a lowering of any critical points [4]. Such phenomenon is explained from the concept of capillary criticality that implies the existence of a temperature, $T_{cc}$, below the bulk critical temperature beyond which liquid-gas phase transition becomes reversible [161].

In the simplest case, the gas-liquid phase transition can be influenced by the presence of a solid substrate. A liquid drop placed on a substrate can either wet it or not, when $\gamma_{sg}=\gamma_{sl}+\gamma_{gl}$ or $\gamma_{sg}<\gamma_{sl}+\gamma_{gl}$, respectively, where $\gamma_{sg}$, $\gamma_{sl}$ and $\gamma_{gl}$ are the substrate-gas, substrate-liquid and gas-liquid surface tensions. A wetting transition occurs when a saturated gas is in contact with a wall. At this transition, a thick liquid layer condenses at the wall while the bulk fluid stays in the gas phase [30]. Similarly, a surface initially covered by a film could be dewetted under an appropriate change of the parameters [31]. Additionally, pre–wetting can also occur if the gas is unsaturated, and there is a formation of a thin liquid layer at the wall [162].

In the presence of an electric field, oppositely charged ions are attracted to the interface between a conductive and a non-conductive (dielectric) material and exert an interfacial force. These induced forces are especially strong as the electric field becomes large. However, due to short-range attractions between ions of the same sign, and the entropy of mixing effect, coions are also attracted together with counterions. If the electric field is sufficiently strong, electrowetting occurs [163].

In confined systems, there are effects derived from the influence of different length scales corresponding to particle sizes, ranges of intermolecular potentials, and dimensions of confinement [158,164]. The most common example of a confinement–induced phase transition in cylindrical or slit-shaped pores is the capillary condensation phenomenon that can occur if the walls attract the particles, and $\gamma_{sl}-\gamma_{sg}<0$. At the capillary condensation, a gas at pressure $p<p_{sat}$ condenses to a liquid-like phase that fills the pore [30]. The shift of the chemical potential at the capillary condensation in a slit of a width *w* is given by the Kelvin equation

(8) $$\mu_{cc}-\mu_{sat}=\frac{2(\gamma_{sl}-\gamma_{sg})}{w(\rho_{l}-\rho_{g})},$$

where $\rho_{l},\rho_{g}$ denote the density of the liquid and the gas phases at the bulk coexistence, respectively.

The amount of the fluid adsorbed in the pore, or the adsorption, is given by the formula

(9) $$\Gamma =\int_{0}^{w}\left(\rho \left(z\right)-\rho_{b}\right)dz$$

where $\rho_{b}$ is the bulk density at given μ. At the capillary condensation phase transition (for $\mu =\mu_{cc}$), Γ jumps between two values, characterizing the gas-like and liquid-like phases in the pore, in analogy to the jump of the density at the phase coexistence in the bulk [30].

When the confining materials are charged, then electrocapillary phenomena take place. Electrocapillarity involves the thermodynamics of charged interfaces and is related to changes in the interfacial energy such as the electrode potential or changes in the concentration of the electrolytes in solution [165]. Electrocapillarity research was started with the work of Gabriel Lippmann [166] who found that changes in voltage influence the capillary depression of mercury in contact with electrolyte solutions [163]. The electrocapillary phenomena can occur at other interfaces as well, for instance, at interfaces between two immiscible electrolyte solutions [165].

Capillary condensation of IL-solvent mixtures in porous electrodes was reported in [167]. A simplified system of a single electrode pore immersed in a bulk electrolyte was considered, and, as a result of approaching capillary condensation, a fluctuation-enhanced capacitance over a range of surface potentials was found. This enhancement is attributed to density fluctuations in the screening electrolyte due to the phase transition. This enhanced capacitance was also found near the critical point. In real electrodes, the pore width is highly inhomogeneous; however, this could be advantageous for inducing fluctuation-enhanced capacitance. This fact relies on the idea that the topology of the pore can be tailored to display optimal capacitance over a specific potential window [167].

Another interesting behavior related to the confinement-induced phase transition is reported in [168]. Tuning-fork-based atomic force microscope measurements revealed a dramatic change of the ILs towards a solid-like phase denoting capillary freezing. This phase transition occurs below a threshold, which is related to the nature of the confining materials; metallic surfaces promote freezing. Such behavior is explained by the fact that confinement shifts the freezing transition, and there is an influence of the electronic screening on IL wetting of the confining surfaces. In supercapacitors, freezing transitions are avoided by using disordered and rough surfaces. However, freezing may be beneficial in lubrication, where the formation of a weak solid phase would prevent an undesired direct substrate–substrate contact [168].

## 5. Effect of Phase Separation on Electrochemical Properties of Confined IL–Solvent Mixture

**Figure 3:**
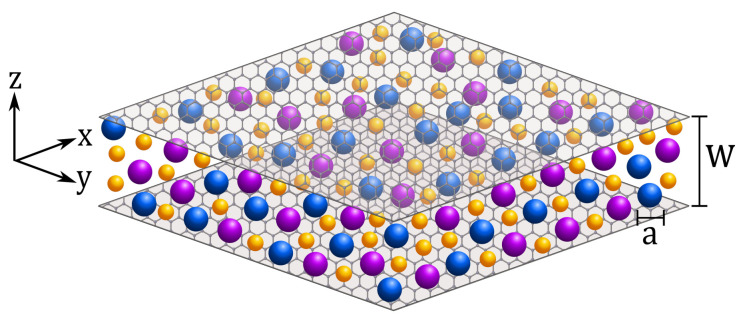
IL-solvent mixture confined in a slit-shaped mesopore. The electrodes are separated by a distance *w*, and the electrostatic potential, *U*, applied at z=0 and z=w is kept constant with respect to the bulk. The ion diameter, denoted by *a*, is the same for cations (blue) and anions (purple spheres). Yellow spheres represent the solvent. The ionophilicity (or surface field) at z=0 and z=w is denoted by *h_s_* and describes the electrode preference for ions or solvent.

Until very recently, only Coulombic and steric interactions were taken into account in the majority of theoretical studies of electrochemical properties of EDL in ILS. However, the specific interactions can play a very significant role as evidenced by the phase separation into IL-poor and IL-rich phases induced by these interactions [156,157]. The effect of the specific interactions on capacitance *C* (see Equation (3)), charge *Q* (see Equation (4)) and stored energy *E* (see Equation (6)) was studied recently in [35,36,37], where the authors have considered a mixture of IL and neutral solvent confined by a slit-shaped mesopore, as shown in Figure 3. Special attention was paid to the proximity of the phase transition. In these works, the Poisson–Boltzmann theory is combined with the mean-field (MF) theory for phase separation in mixtures, and the grand thermodynamic potential has the following form [35,36]

(10) $$\begin{array}{cc} \beta \;\Omega [\rho_{\pm },u]/A & =\int_{0}^{}\left[\rho_{+}\ln\left(a^{3}\rho_{+}\right)+\rho_{-}\ln\left(a^{3}\rho_{-}\right)+\beta f_{ex}\left(\rho \right)\right]dz\\ \; & +\int_{0}^{}\left[cu-\frac{1}{8\pi \lambda_{B}}{\left(\frac{\partial u}{\partial z}\right)}^{2}\right]dz\\ \; & +\beta K\left\{\int_{0}^{}\left[\frac{\xi_{0}^{2}}{2}{\left(\frac{\partial \rho }{\partial z}\right)}^{2}-\frac{1}{2}\rho^{2}\right]dz+\frac{\xi_{0}}{2}\rho_{0}^{2}{-}_{0}+\frac{\xi_{0}}{2}\rho_{}^{2}{-}_{}\right\}\\ \; & -\beta \mu \int_{0}^{}\rho dz \end{array}$$

where $\beta =1/(k_{B}T)$, is the slit width, *A* the surface area, $\rho_{\pm }$ are the cation and anion densities and μ is the chemical potential difference between the ions and the solvent. The total ion density is $\rho =\rho_{+}+\rho_{-}$, and the charge density is $c=\rho_{+}-\rho_{-}$ (in units of the elementary charge *e*). In the case of a single planar electrode, w→∞.

The first term in Equation (10) is the entropic contribution where the first two terms come from the entropy of mixing of ions, and the last term corresponds to the excess free energy associated with the excluded volume interactions, $f_{ex}$. In [35,37], the Carnahan–Starling (CS) approximation [169] for the excluded volume interactions between the ions was considered,

(11) $$\begin{array}{c} \beta f_{ex}^{CS}\left(\rho \right)=\rho \left(\frac{4\eta -3\eta^{2}}{{\left(1-\eta \right)}^{2}}-1\right), \end{array}$$

where $\eta =\pi \rho a^{3}/6$ is the packing fraction of ions. In [36], the CS approximation was compared with the popular lattice-gas expression

(12) $$\begin{array}{c} \beta f_{ex}^{lg}\left(\rho \right)=\left(\rho_{tot}-\rho \right)\ln\left[a^{3}\left(\rho_{tot}-\rho \right)\right], \end{array}$$

which is derived from the solvent’s ideal-gas entropy, $\beta f_{ex}=\rho_{s}\ln a^{3}\rho_{s}$, by assuming the local incompressibility condition, $\rho_{+}\left(r\right)+\rho_{-}\left(r\right)+\rho_{s}\left(r\right)=\rho_{tot}$ ($\rho_{tot}=a^{-3}$ for the lattice–gas model). Equation (12) has been implemented in various relevant studies, most notably by Bikerman [106], Wicke and Eigen [107,109], Borukhov et al. [52], Kilic et al. [113] and Kornyshev [54].

The second term in Equation (10) corresponds to the electrostatic energy in $k_{B}T$ units [52,170,171,172], where *u* is the electrostatic potential, and $\lambda_{B}=\beta e^{2}/\epsilon$ is the Bjerrum length, with ϵ assumed to be temperature- and position-independent. With the assumption of constant ϵ, the theory cannot be accurate on the quantitative level but should capture generic effects and qualitative behavior. The first two terms in (10) represent the well-known theory for the electrolyte with neglected specific interactions.

The third term in Equation (10) is the contribution from attractive non-Coulombic van der Waals-like interactions, which may lead to demixing of the IL and solvent. When the phase separation is promoted by the chemical difference between IL and neutral solvent, one can take into account only the effective interactions leading to the phase separation [171,173]. The parameter *K* measures the strength of the interactions and sets a temperature (energy) scale expressed via the bulk system’s critical temperature ${\bar{T}}_{c}=k_{B}T_{c}a^{3}/K$. The parameter $\xi_{0}∼a$ is interaction’s spatial extension [35]. The electrode’s ionophilicity is denoted by *h_s_* and describes the electrode’s preference for ions or solvent; *h_s_* > 0 means that the wall favors ions. This preference was assumed to be the same for anions and cations.

**Figure 4:**
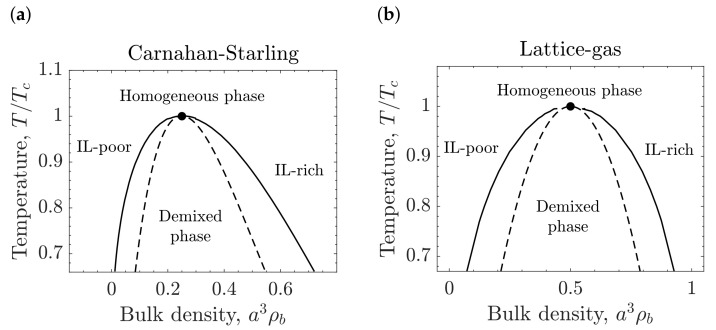
Bulk phase diagrams of ionic liquid (IL)-solvent mixtures. (**a**) Excluded volume given by the Carnahan-Starling approximation. (**b**) Excluded volume given by the lattice-gas approximation. The solid lines represent the first order phase transition between the homogeneous and IL-solvent demixed phases, and the dashed lines are the spinodal curves. The circles denote (upper) critical points. Temperature is expressed in terms of the critical temperature $T_{c}$.

In the absence of confining surfaces, c=0 and the bulk ion density $\rho_{b}$ is position independent. In this case Equation (10) simplifies to

(13) $$\beta \Omega_{b}\left({\bar{\rho }}_{b}\right)/V=-\beta K\frac{{\bar{\rho }}_{b}^{2}}{2}+{\bar{\rho }}_{b}\ln\left(\frac{{\bar{\rho }}_{b}}{2}\right)+\beta f_{ex}\left({\bar{\rho }}_{b}\right)-\beta \mu {\bar{\rho }}_{b}.$$

where V=A is the volume, and ${\bar{\rho }}_{b}=a^{3}\rho_{b}$. The homogeneous IL-solvent mixtures becomes unstable with respect to density fluctuations at the spinodal line, $\partial^{2}\Omega_{b}/\partial {\bar{\rho }}_{b}^{2}=0$, shown as the dashed lines in Figure 4. The critical point (shown by solid circles in the same figure) corresponds to the point on the spinodal that satisfies $d{\bar{T}}_{c}\left({\bar{\rho }}_{b}\right)/d{\bar{\rho }}_{b}=0$. For the CS and lattice-gas expressions, the critical density of ions and temperature are ${\bar{\rho }}_{c}\approx 0.25$, ${\bar{T}}_{c}\approx 0.09$ and ${\bar{\rho }}_{c}\approx 0.5$, ${\bar{T}}_{c}\approx 0.25$, respectively. For $\bar{T}<{\bar{T}}_{c}$, IL-rich and IL-poor phases appear, and the coexistence line between them (solid lines in Figure 4) was found by numerical solution of the equations $\partial \Omega_{b}/\partial {\bar{\rho }}_{b}=0$ and $\Omega_{b}\left({\bar{\rho }}_{rich}\right)=\Omega_{b}\left({\bar{\rho }}_{poor}\right)$, where ${\bar{\rho }}_{rich}$ and ${\bar{\rho }}_{poor}$ denote dimensionless densities in the IL-rich and IL-poor phases, respectively.

The model presented above is suitable for IL-solvent mixtures that exhibit phase separation induced by the van der Waals interactions, for example, for aqueous bistriflimide (TFSI)-based ILs or for 1-hexyl-3-methylimidazolium tetrafluoroborate (C6mim-BF4) in alcohol (C6OH). Confinement effects discussed in the following sections concern, in particular, the above systems.

### 5.1. Il-Solvent Mixtures in Contact with a Single Electrode

**Figure 5:**
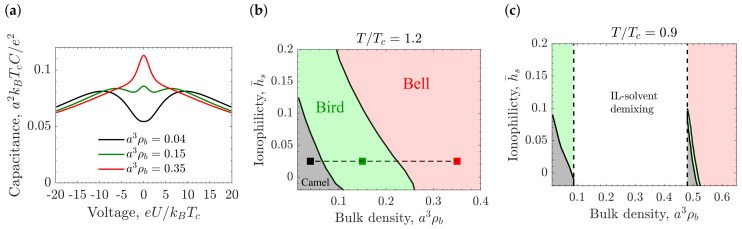
Differential capacitance close to demixing at constant temperature. (**a**) Differential capacitance for the Carnahan–Starling (CS) approximation as a function of applied potential at constant temperature, $T/T_{c}=1.2$, ionophilicity ${\bar{h}}_{s}=a^{3}h_{s}/\xi_{0}=0.025$, and three different bulk densities, demonstrating the camel-, bird-, and bell-shaped capacitance. (**b**) Capacitance diagram for the Carnahan-Starling (CS) approximation showing the regions of camel, bird, and bell-like capacitance at constant temperature, $T/T_{c}=1.2$. The dashed horizontal line denotes the value of ${\bar{h}}_{s}=a^{3}h_{s}/\xi_{0}=0.025$ and the symbols mark the bulk densities $\rho_{b}$ used in panel (**a**). (**c**) Capacitance diagram for the Carnahan-Starling (CS) approximation showing the regions of camel, bird, and bell-like capacitance for temperature below $T_{c}$, $T/T_{c}=0.9$. The white region denotes the domain of the IL-solvent demixing (Figure 4a). For common values of the ion diameter a=0.7 nm and room temperature for $T_{c}$, the various units are: thermal voltage $e/k_{B}T_{c}$ ≈ 26 mV for voltage, thermal electric capacitance $e^{2}/\left(k_{B}T_{c}a^{2}\right)\approx 620$ μFcm^−2^ for capacitance.

The equilibrium charge and density profiles, c(z) and ρ(z) respectively, near a planar electrode located at z=0 correspond to the minimum of Ω defined in Equation (10), with →∞. The differential Euler–Lagrange equations following from the minimization of Equation (10), and the Poisson equation were solved numerically in [35,36] in the one-phase region just above the demixing. Figure 5a shows that the differential capacitance (see Equation (3)) can have multiple peaks as a function of the applied potential *U*, depending on the IL concentration $\rho_{b}$. In the case of low ion concentrations, the capacitance curve exhibits the camel-like shape with a minimum at the PZC and two symmetric maxima, as expected [49,50,51,54,56,57,59,115,116,174]. At higher concentrations, the well-studied bell-shaped capacitance that exhibits a single maximum at the PZC was obtained. At intermediate IL concentrations, however, the capacitance can exhibit three peaks. This new shape has been named "bird-like" capacitance [35,36] because of the similarity of its shape to a flying bird. The lattice-gas approximation also predicts the bird-shaped capacitance. Experimentally, the emergence of humps at the PZC in the U-shaped capacitance was reported for N_2_-saturated room-temperature ILs on some electrodes Alam et al. [174]; the appearance of wings in the bell-shaped capacitance was obtained from a simulation study by Sha et al. [124] for neat BMIM-PF_6_ on a gold surface.

**Figure 6:**
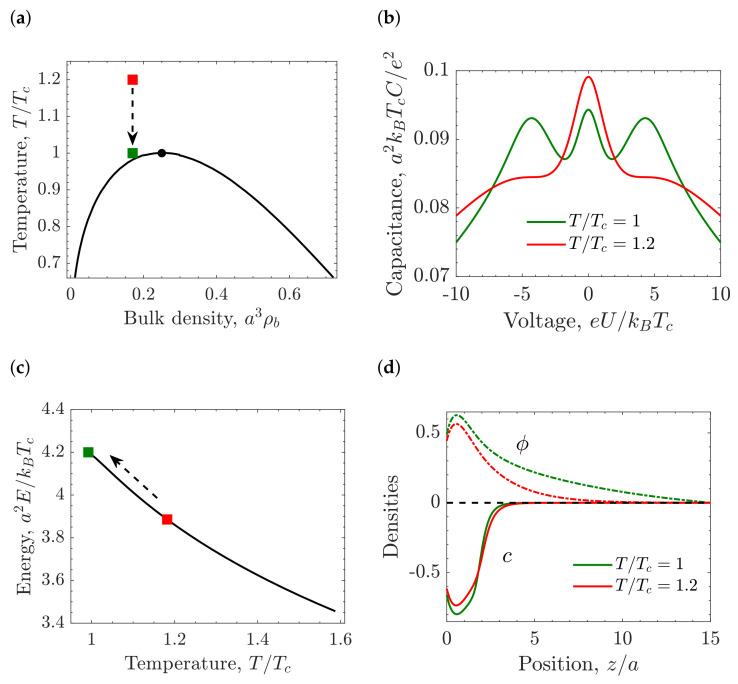
Differential capacitance close to demixing for the Carnahan–Starling (CS) approximation. (**a**) Bulk phase diagram in the temperature-bulk density plane showing the temperatures used in the following panels. The red square denotes $T/T_{c}=1.2$, whereas the green square represents $T/T_{c}=1$. (**b**) Differential capacitance as a function of applied potential at constant bulk density $\rho_{b}=0.17$, ionophilicity ${\bar{h}}_{s}=a^{3}h_{s}/\xi_{0}=0.08$, and two different temperatures, demonstrating the bell-shaped capacitance for $T/T_{c}=1.2$ and the bird-shaped capacitance for $T/T_{c}=1$. (**c**) Stored energy as a function of temperature. (**d**) Density profiles. Order parameter $\phi =a^{3}\left(\rho -\rho_{b}\right)$ (dashed lines), where $\rho =\rho_{+}+\rho_{-}$ is the ion density, and the charge density $c=a^{3}\left(\rho -\rho_{b}\right)$ (solid lines) calculated at $T/T_{c}=1$ (green lines) and $T/T_{c}=1.2$ (red lines) marked by colored squares in the previous panel. The bulk density $\rho_{b}=0.17$, voltage $eU/k_{B}T_{c}=20$, and ionophilicity ${\bar{h}}_{s}=a^{3}h_{s}/\xi_{0}=0.08$. For common values of the ion diameter *a* = 0.7 nm and room temperature for *T_c_*, the various units are: thermal voltage $e/k_{B}T_{c}$ ≈ 26 mV for voltage, thermal electric capacitance $e^{2}/\left(k_{B}T_{c}a^{2}\right)\approx 620$ μFcm^−2^ for capacitance.

Figure 5b,c present the capacitance diagram in the ionophilicity-bulk density plane for temperatures above and below the critical temperature, respectively. Interestingly, the CS approximation predicts the camel-like shape even for high densities if the electrode is strongly ionophobic. Such behavior is explained by the fact that an ionophobic electrode drives the formation of a thick near-electrode layer of an ion-poor phase. Changes in the temperature at fixed bulk density can also induce a transformation from the bell-shaped capacitance at high *T* to the bird-like capacitance at lower *T*, as is shown in Figure 6a,b.

Close to demixing, the voltage-induced ion density increases at the surface vicinity. As a consequence, the capacitance increases in such a way that the bell-shaped capacitance acquires wings, and the bird-shaped capacitance emerges (Figure 6b). Figure 6c shows the stored energy as a function of the temperature. The stored energy increases when the temperature decreases, i.e., there is an energetic gain while the system approaches demixing. Electricity generation from waste heat could be a possible application coming from the temperature dependence of the stored energy [13,14,175,176,177]. Figure 6d shows the order parameter, $\phi \left(z\right)=\bar{\rho }\left(z\right)-{\bar{\rho }}_{b}$, and charge density profiles. At temperature $T/T_{c}=1$, the order parameter grows next to the surface because the dispersion interactions favor higher ion densities near the ionophilic electrode, partially compensating the electrostatic repulsion of the coions. Therefore, the stored energy and the accumulated charge are higher for decreasing temperature.

Concerning the lattice-gas approximation, the conversion between the different capacitance shapes are shifted to higher densities. This trend is consistent with the bulk phase diagram, in which the demixing region and the critical point are also moved to higher densities (Figure 4b) [35,36].

### 5.2. Capillary Ionization and Charging of Slit Mesopores

Confinement-induced phase transitions, especially the capillary condensation, were intensively studied for decades [29,30,160,178]. In IL-solvent mixtures confined in slit mesopores, a *capillary ionization* that is analogous to the capillary condensation can occur. At the capillary ionization transition, the IL-rich phase condenses in the pore for the chemical potential of ions corresponding to the stability of the IL-poor phase in bulk. The studies of the effect of the capillary ionization on electrochemical properties of IL-solvent mixtures confined in mesopores, however, started only very recently [37]. In [37], the grand potential functional Equation (10) with finite distance *w* between parallel electrodes was considered. It was shown that the capillary ionization transition could be induced by changing parameters such as temperature, slit width (*w*), ionophilicity (*h_s_*), and potential difference (*U*) applied to the pore walls.

**Figure 7:**
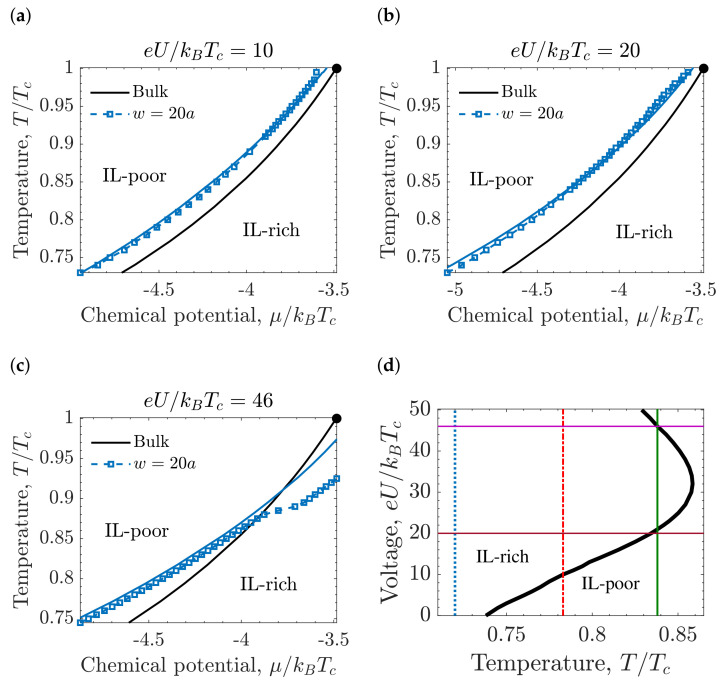
Capillary ionization of slit mesopores under applied voltages. (**a**–**c**) Phase diagram plotted in the temperature-chemical potential plane for (**a**) $eU/k_{B}T_{c}=10$, (**b**) $eU/k_{B}T_{c}=20$, (**c**) $eU/k_{B}T_{c}=46$. The symbols have been obtained by numerical calculations, and the solid blue line corresponds to the results of the Kelvin equation. The solid black line is the phase coexistence in bulk, and the black dot is the critical point. (**d**) Phase diagram plotted in the voltage-temperature plane for the chemical potential $\mu /k_{B}T_{c}=-4.57$. The thick black line denotes the first-order transitions between the IL-rich and IL-poor phases while the thin vertical lines indicate the temperatures: $T/T_{c}=0.72$ (blue), $T/T_{c}=0.78$ (red), $T/T_{c}=0.838$ (green), used in Figure 9. The horizontal lines mark the values of voltage used in Figure 10. The slit width w=20a, where *a* is the ion diameter, and the ionophilicity $a^{3}h_{s}/\xi_{0}=0.25$, where $\xi_{0}$ is the bare correlation length.

**Figure 8:**
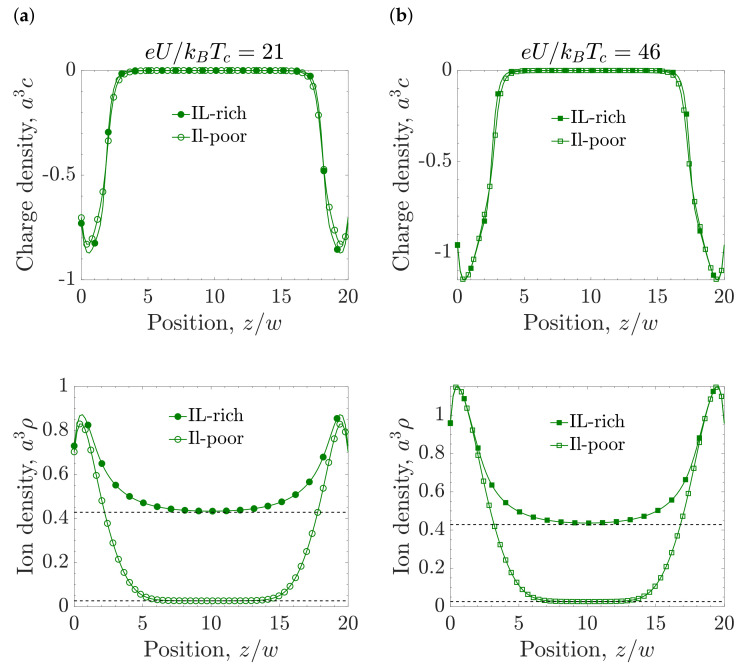
Charge and ion density profiles at the capillary ionization transition. (**a**) Charge and ion density profiles at $eU/k_{B}T_{c}=21$. (**b**) Charge and ion density profiles at $eU/k_{B}T_{c}=46$. In all the plots, the chemical potential $\mu /k_{B}T_{c}=-4.57$, the temperature $T/T_{c}=0.838$, slit width w=20a, and ionophilicity $a^{3}h_{s}/\xi_{0}=0.25$, where $\xi_{0}$ is the bare correlation length and *a* the ion diameter. The dashed horizontal lines show the bulk values in the corresponding phases. The filled symbols represent the IL-rich phase while the unfilled symbols denote the IL-poor phase.

Figure 7a–c show the phase diagrams plotted in the temperature-chemical potential plane for different applied voltages, and Figure 7d shows the phase diagram plotted in the voltage-temperature plane. Except at very high voltages, good agreement between the results from the Kelvin Equation (8) and the numerical minimization of Equation (10) was obtained. From Figure 7a,b, it follows that as the applied voltage increases, the stability region of the IL-rich phase becomes wider. The increasing region of the stability of the IL-rich phase under applied voltage can be seen in Figure 7d too, but only up to $eU/k_{B}T_{c}=32$. Counterintuitively, at high voltages, the transition line moves back to larger chemical potentials (Figure 7c), and the stability region of the IL-poor phase increases. The increasing stability region of the IL-poor phase for increasing voltage is clearly seen in Figure 7d for $eU/k_{B}T_{c}>32$, since the transition curve bends at $eU/k_{B}T_{c}=32$. This implies that the capillary deionization transition occurs (from the IL-rich phase to the IL-poor phase) for increasing voltage at fixed *T* for $eU/k_{B}T_{c}>32$. Such re-entrant behavior of the capillary ionization follows from the near-electrode structure. From the charge and ion density profiles (Figure 8) one can see that the charge and density near the electrode are larger in the IL-rich phase when the voltage is not large, and the electrostatic and wall-fluid potentials stabilize this phase. At high voltage (Figure 8b), the charge density and the ion density near the pore walls are the same in both phases. Thus, the thermodynamic state becomes determined by the in-pore bulk region, favoring the IL-poor phase for the considered μ.

**Figure 9:**
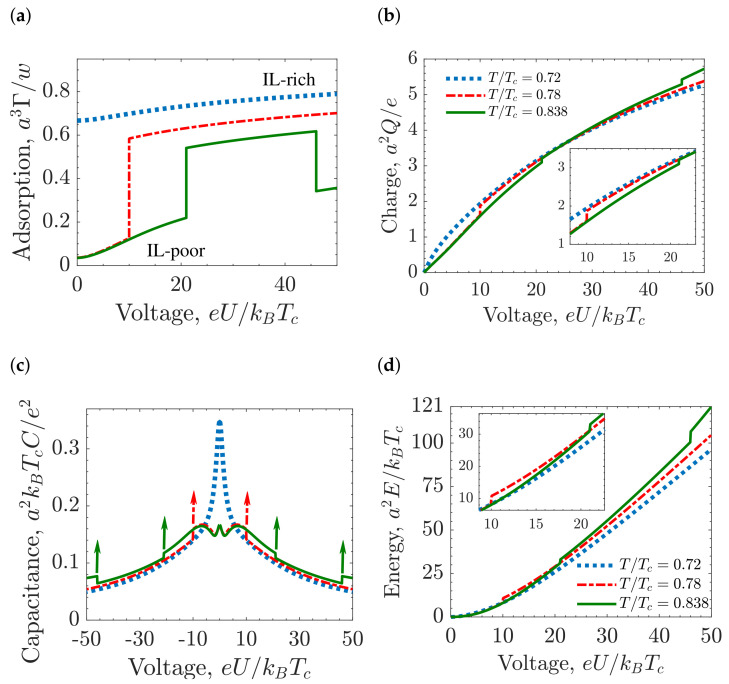
Voltage-induced capillary ionization and charging of the slit mesopores. (**a**) Amount of IL adsorbed in the pore, Γ (see Equation (9)). (**b**) Accumulated charge in the pore as a function of the applied voltage. (**c**) Differential capacitance. The vertical arrows indicate that capacitance diverges at the capillary ionization transition. (**d**) Stored energy. In all plots the pore width w=20a, chemical potential $\mu /k_{B}T_{c}=-4.57$ and ionophilicity $a^{3}h_{s}/\xi_{0}=0.25$, where *a* the ion diameter, and $\xi_{0}$ is the bare correlation length. For common values of the ion diameter *a* = 0.7 nm and room temperature for $T_{c}$, the various units are: thermal voltage $e/k_{B}T_{c}$ ≈ 26 mV for voltage, $e/a^{2}$ ≈ 2 *e*nm^−2^ ≈ 32 μCcm^−2^ for accumulated charge, thermal electric capacitance $e^{2}/\left(k_{B}T_{c}a^{2}\right)$ ≈ 620 μFcm^−2^ for capacitance, and $k_{B}T_{c}/a^{2}$ ≈ 0.84 mJcm^−2^ ≈ 0.23 nWcm^−2^ for energy.

Figure 9a,b show the ion adsorption Γ (see Equation (9)) and the accumulated charge *Q* (see Equation (4)), respectively, calculated at three different temperatures. If there is no transition ($T/T_{c}=0.72$), Γ and *Q* are both continuous functions of voltage. At $T/T_{c}=0.78$, a capillary phase transition occurs at $eU/k_{B}T_{c}=10$, and there is a jump in Γ that represents the transition from the IL-poor to IL-rich phase. At $T/T_{c}=0.838$, Γ exhibits two transitions, from the IL-poor to IL-rich phase at $eU/k_{B}T_{c}=21$, and the other one from IL-rich to IL-poor phase at $eU/k_{B}T_{c}=46$.

Capillary ionization transition is accompanied by an abrupt increase of the charge accumulated in the pore. This fact has important consequences for the capacitance (Figure 9c) and the energy storage (Figure 9d). At lower temperatures, the system is in the IL-rich region, characterized by a high ion density, and the capacitance has a bell shape, consistent with earlier studies [54]. At high temperatures, the IL-poor phase becomes stable, and the capacitance shape changes to bird-like [35,111]. The capacitance at the capillary ionization diverges; the vertical arrows in Figure 9c for the temperatures $T/T_{c}=0.78$ and $T/T_{c}=0.838$ denote this divergence, meaning that the capacitance tends to infinity at the capillary ionization transition. Figure 9d corresponds to the stored energy in the pore, and one can see that there is an additional contribution at the transition given by $\Delta E_{ci}=U_{ci}\Delta Q_{ci}$, which appears as a jump in the stored energy.

**Figure 10:**
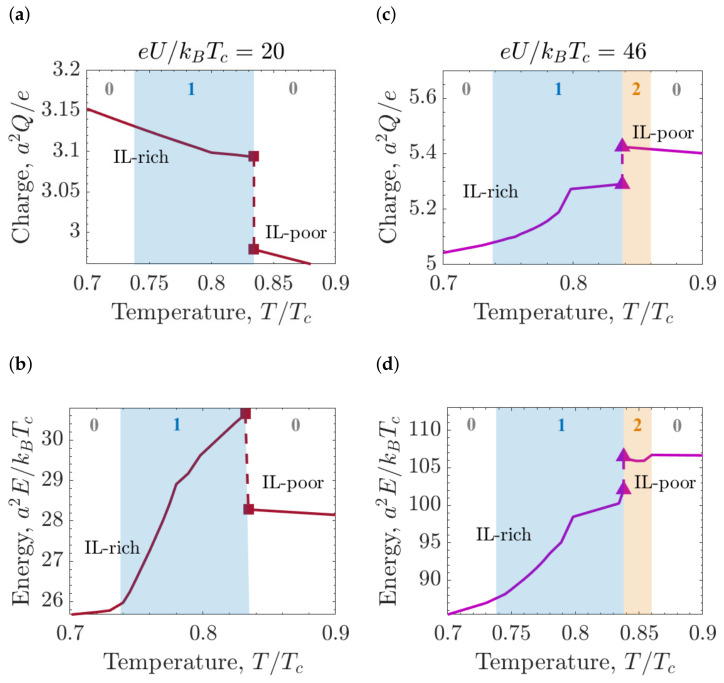
Accumulated charge and stored energy in slit mesopores. (**a**) Accumulated charge for applied voltage $eU/k_{B}T_{c}=20$. (**b**) Stored energy for applied voltage $eU/k_{B}T_{c}=20$. (**c**) Accumulated charge for applied voltage $eU/k_{B}T_{c}=46$. (**d**) Stored energy for applied voltage $eU/k_{B}T_{c}=46$. In all the panels, chemical potential $\mu /k_{B}T_{c}=-4.57$, slit width w=20a and ionophilicity $a^{3}h_{s}/\xi_{0}=0.25$, where $\xi_{0}$ is the bare correlation length and *a* the ion diameter.

The charge and stored energy in a pore as a function of temperature, calculated at low and high voltages, are shown in Figure 10. The jumps of the charge and energy at the capillary ionization transition are positive when the transition line is crossed for increasing *U* (Figure 9d). At low voltages, the transition proceeds from the IL-poor to the IL-rich phase upon increasing *U* or decreasing *T*, while at high voltages, the transition proceeds from the IL-rich to the IL-poor phase when *U* and/or *T* increases (Figure 7d). Since the jumps of *Q* and *E* at the transition are positive for increasing *U*, when the transition line is crossed by increasing *T* at constant *U*, these jumps are negative at low *U*, and positive at high *U*.

**Figure 11:**
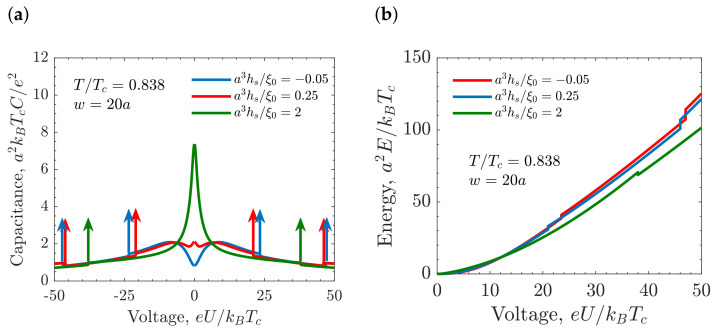
Influence of the ionophilicity on the capacitance and the stored energy of equally charged slit-shaped mesopore. (**a**) Differential capacitance. The vertical arrows indicate that capacitance diverges at the capillary ionization transition. (**b**) Stored energy in the pore as a function of the applied voltage. In all the plots, the chemical potential $\mu /k_{B}T_{c}=-4.57$, the slit width w=20a, and temperature $T/T_{c}=0.838$. The blue lines correspond to ionophilicity $a^{3}h_{s}/\xi_{0}=-0.05$ (ionophobic electrodes), red lines to $a^{3}h_{s}/\xi_{0}=0.25$ (mild-ionophilic electrodes), and green lines to $a^{3}h_{s}/\xi_{0}=2$ (strongly ionophilic electrodes); *a* is the ion diameter and $\xi_{0}$ the bare correlation length. For common values of the ion diameter *a* = 0.7 nm and room temperature for $T_{c}$, the various units are: thermal voltage $e/k_{B}T_{c}$ ≈ 26 mV for voltage, thermal electric capacitance $e^{2}/\left(k_{B}T_{c}a^{2}\right)$ ≈ 620 μFcm^−2^ for capacitance, $k_{B}T_{c}/a^{2}$ ≈ 0.84 mJcm^−2^ ≈ 0.23 nWcm^−2^ for energy, and $e/a^{2}$ ≈ 2 *e*nm^−2^ ≈ 32 μCcm^−2^ for accumulated charge.

The effect of the electrode’s ionophilicity on the stored energy and the capacitance is shown in Figure 11, where the capacitance and the stored energy are shown as functions of *U*. Strongly ionophilic electrodes display the bell-shaped capacitance due to the high amount of ions near the surface. The capacitance decreases for increasing voltage because of the screening of the electrode surface by the dense ionic layer. An ionophobic electrode displays the camel-shaped capacitance that exhibits a minimum at PZC and a maximum at higher voltages that favor the ion migration towards the electrode surface. Medium-ionophilic electrodes display the bird-shaped capacitance. At high voltages, the differential capacitance of ionophobic electrodes is higher compared to ionophilic ones. As a result, the stored energy at high voltages is higher for the ionophobic electrode (Figure 11b).

The theory developed in [35,36,37] does not take into account microscopic details and fluctuations. The results, however, show interesting physics following from mutual effects of thermodynamic and electrochemical properties. The observed phenomena are not only interesting from the fundamental point of view but may find practical applications and are worth further investigation by more accurate theories, simulations, and experiments.

## 6. Conclusions

In this review, we have shown that despite decades of intensive studies of ionic systems, unexpected and surprising properties and phenomena can still be discovered. This concerns, in particular: (i) structural properties and patterns formed by ions near electrodes, (ii) effects of confinement, in particular the dependence of the disjoining pressure between confining plates on the concentration of ions in IL-solvent mixtures (iii) electrical double-layer capacitance, stored energy and charge, especially near the demixing phase transition in IL-solvent mixtures and (iv) capillary ionization transition in porous electrodes and its effect on capacitance, energy and charge. On the one hand, there is a need for a theoretical explanation of experimental results that contradict classical theories, and on the other hand, there are theoretical predictions that need to be verified by simulations and experiments. The first case concerns, in particular, the structure and mechanical properties of confined IL-solvent mixtures. The second case concerns the theoretical predictions of the effects of phase transitions on electrochemical properties of IL-solvent mixtures near a single electrode and in slit-shaped pores. These predictions have been confirmed in simulation studies of a coarse-grained model of IL-solvent mixture [179], but still require verification by atomistic simulations of realistic models and by experiment. We have shown that there still remain open questions that are of fundamental and practical importance. IL and IL-solvent mixtures remain a vital and fascinating subject.

## Data Availability

The data presented in this study are available on request from Carolina Cruz (ccruz@ichf.edu.pl).

## Abbreviations

The following abbreviations are used in this manuscript:

IL	Ionic Liquid
EDL	Electrical double layer
PB	Poisson-Boltzmann
GCS	Gouy-Chapman-Stern
EW	Electrochemical window
PZC	Potential of zero charge
CS	Carnahan-Starling
ci	capillary ionization
APILs	Aprotic ionic liquids
PILs	Protic ionic liquids
